# Tailored CAMHS Educational Sessions for Primary Care Staff

**DOI:** 10.1192/bjo.2024.326

**Published:** 2024-08-01

**Authors:** Anna Sherratt, Michael Foster

**Affiliations:** 1 Midlands Partnership NHS Foundation Trust, Telford, United Kingdom; 2 North Staffordshire Combined Healthcare NHS Trust, Stoke-on-Trent, United Kingdom

## Abstract

**Aims:**

To develop and evaluate a tailored teaching session for local non-medical primary care staff on common CAMHS (Child and Adolescent Mental Health) conditions. It was hypothesised that quizzes administered before and after the educational session would evidence an improvement in clinician's knowledge of these clinical presentations.

**Methods:**

Invitations were extended to all local PCNs to attend educational sessions held on four separate occasions in December 2022 and January 2023. Multiple choice quizzes were administered before and after a presentation on four common CAMHS conditions. The presentation and quizzes covered the presentation, diagnosis, and management of autism, eating disorders, depression and emotional dysregulation. Quizzes were scored out of a maximum 16 points with four questions per clinical condition. A paired T-test (following tests for normal variance) was performed using JASP software to compare the before and after scores.

**Results:**

A total of 22 non-medical clinical staff attended the sessions. This included physician associates (n = 1), allied health professionals (n = 5), practice nurse (n = 3), care coordinator (n = 3), health care assistant (n = 4), social prescriber (n = 1), mental health practitioner (n = 3), advanced clinical practitioner (n = 1) and advanced nurse practitioner (n = 1). For the 22 pairs of quizzes, mean differences and 95% confidence intervals (CIs) were calculated between before-and-after scores. The mean difference between total score was 6.9 CI [6.1, 7.7] which was statistically significant (p < 0.001).

**Conclusion:**

More than 31,000 additional staff have been recruited into healthcare roles at general practices across the country since 2019 to meet soaring demand for primary care services. Since the pandemic record numbers of children and adolescents are presenting with mental health difficulties, therefore, it is likely that primary care clinicians will encounter these presentations in their practice. Our results suggest that such a tailored approach can offer effective means in improving knowledge in this growing group of professionals. Such sessions may also provide informal spaces in which to network with secondary mental health professionals, improving links between services.

